# Cholinergic Senescence in the Ts65Dn Mouse Model for Down Syndrome

**DOI:** 10.1007/s11064-022-03659-0

**Published:** 2022-06-29

**Authors:** Martina Kirstein, Alba Cambrils, Ana Segarra, Ana Melero, Emilio Varea

**Affiliations:** Cell Biology Department, Universitat de València, Dr. Moliner, 50, Burjassot, 46100 València, Spain

**Keywords:** Cholinergic neurons, Ts65Dn, Down syndrome, Senescence, c-fos, Premature aging, Muscarinic receptors

## Abstract

Down syndrome (DS) induces a variable phenotype including intellectual disabilities and early development of Alzheimer’s disease (AD). Moreover, individuals with DS display accelerated aging that affects diverse organs, among them the brain. The Ts65Dn mouse is the most widely used model to study DS. Progressive loss of cholinergic neurons is one of the hallmarks of AD present in DS and in the Ts65Dn model. In this study, we quantify the number of cholinergic neurons in control and Ts65Dn mice, observing a general reduction in their number with age but in particular, a greater loss in old Ts65Dn mice. Increased expression of the m1 muscarinic receptor in the hippocampus counteracts this loss. Cholinergic neurons in the Ts65Dn mice display overexpression of the early expression gene c-fos and an increase in the expression of β-galactosidase, a marker of senescence. A possible mechanism for senescence induction could be phosphorylation of the transcription factor FOXO1 and its retention in the cytoplasm, which we are able to confirm in the Ts65Dn model. In our study, using Ts65Dn mice, we observe increased cholinergic activity, which induces a process of early senescence that culminates in the loss of these neurons.

**Supplementary Information:**

The online version contains supplementary material available at 10.1007/s11064-022-03659-0.

## Introduction

Down syndrome (DS) is the most common chromosomal alteration with an incidence of one in 1000 live births [1]. Trisomy of human chromosome 21 induces a phenotype that includes immune deficiencies, heart defects, intellectual disability, altered motor development, reduced muscle tone and early development of Alzheimer’s disease (AD) [2–6]. Moreover, and possibly related to AD, individuals with DS display accelerated aging that affects diverse organs, among them the brain [7].

Several animal models that mimic the alterations in DS are available, among them the most widely used is the Ts65Dn mouse [8]. This model is segmentally trisomic for a portion of the mouse chromosome 16, an orthologous to the long arm of the human chromosome 21, which codes for approximately 140 genes, many of which are highly conserved between mice and humans [7, 9, 10]. The phenotype of the Ts65Dn mouse includes alterations in working memory, deficits in long-term memory, motor dysfunctions, reduced pain responsiveness and hyperactivity [7, 11, 12]. Other remarkable features on the cellular level are cholinergic neuron loss related to age, reduced neuronal number in hippocampal regions and imbalance between excitation and inhibition [13–17]. In particular, the progressive loss of cholinergic neurons, which is observed in some studies at 6 months of age in trisomic mice, is linked to cognitive and physiological impairments, one of the neuropathological hallmarks of AD [7, 18, 19].

Post-mortem analysis of samples from AD patients has revealed a significant reduction in the population of cholinergic neurons, giving thus rise to the cholinergic hypothesis for the cognitive decline observed in AD [20]. Now it is postulated that the cholinergic signalling system plays a critical role in the central nervous system by modulating cognitive function, learning and memory in addition to circadian rhythms [21]. Studies in post-mortem samples from old DS individuals have showed the presence of similar alteration between AD and DS [22, 23].

The above mentioned decline in cholinergic neuron number can be also found in the Ts65Dn mouse model and is thought to be related to a decline in processing of pro Nerve Growth Factor (NGF) into mature NGF in aged trisomic mice [7, 24, 25]. Moreover, an increased level of amyloid precursor protein (APP) is attributed to a disruption of NGF transport in cholinergic neurons [26]. All these events generate a profound deficit in the level of this important neuronal growth factor leading to cholinergic neuron apoptosis [25, 27].

A premature mortality rate in individuals with DS as compared to the general population is the result of premature aging [28, 29]. Early aging also affects the brain and is enhanced by the early development of AD due to an excess of amyloid peptide resulting from the presence of an extra copy of the APP gene in DS [29]. The brains of patients with AD present characteristic neurofibrillary tangles and amyloid plaques, however in DS patients, amyloid deposits are already present during adolescence and are increased progressively with age. Amyloid deposits in the brain induce oxidative stress and neuroinflammation leading to synaptic dysfunction and neuronal death, thus accelerating disease progression [30]. Oxidative stress as a possible cause of neurodegeneration has been widely analysed in DS. Noteworthy, more than 10 genes in the human chromosome 21 codify for proteins related to oxidative stress. Recent studies of our group show an increase of molecules related to oxidative stress such as COX-2 in the temporal cortex of Ts65Dn mice and humans with DS [31]. Dysregulation of other molecules such as reelin that is present in AD, is observed early in life in DS individuals and the Ts65Dn mice model [32].

Although the loss of cholinergic neurons with age is clearly documented in neuropathological diseases, controversy exists about the timely onset of the loss [33]. In this study, we analyse the temporal process of cholinergic neuron loss, the changes in cellular function and their terminal targets. Investigating the possible mechanism of neuronal cell death, we demonstrate the pivotal role of the pAKT/pFOXO1 pathway as a main modulator of these effects.

## Experimental Procedures

Experimental mice were generated by repeated backcrossing of Ts65Dn females to C57/6Ei x C3H/HeSnJ (B6EiC3) F1 hybrid males. The parental generation was obtained from the research colony of Jackson Laboratory. Euploid littermates of Ts65Dn mice served as controls. For this study, we used 36 male mice (18 trisomic mice and 18 euploid mice) of three different ages, 1, 6 and 14 month-old (6 each). The genotypic characterization was established by qRT-PCR using SYBR Green PCR master mix (applied biosystems) from genomic DNA extracted of mice tails by means of the phenol–chloroform method. The relative amount of each gene was quantified by the ABI PRISM 7700 (Applied Biosystems). The genes analysed where APP (3 copies) and Apo-B (2 copies) as previously described [34, 35]. The primers used were the following: for APP (APP-F 5ʹ-TGT TCG GCT GTG TGA TCC TGT GAC-3ʹ; APP-R 5ʹ-AGA AAC GAG CGG CGA AGG GC-3ʹ) and for Apo-B (Apo-B-F 5ʹ-TGC CAG GCT TGT GCT GCT GT-3ʹ; Apo-B-R 5ʹ-GGG TGC TGC CTT TCT CTT GGG G-3ʹ). All animal experimentation was conducted in accordance with the Directive 2010/63/EU of the European Parliament and of the Council of 22 September 2010 on the protection of animals used for scientific purposes and was approved by the Committee on Bioethics of the Universitat de València. Every effort was made to minimize the number of animals used and their suffering. Animals were housed under a normal 12 h light/12 h dark cycle. Water and food were available ad libitum.

The animals were kept in isolated boxes the last 24 h before the moment of sacrifice (kept in a 12 h light/dark schedule) in order to prevent c-fos expression alterations. Then, under pentobarbital overanesthesia (intraperitoneally, 120 mg/kg), the animals were transcardially perfused using a saline solution followed by a solution containing 4% paraformaldehyde in PB (0.1 M, pH 7.4). Brains were removed and cryoprotected using 30% sucrose. Fifty microns Sects. (6 subseries for each brain) were obtained using a sliding freezing microtome.

## Immunohistochemical Procedure (DAB)

Tissue was processed “free-floating” for immunohistochemistry as follows. Briefly, sections were incubated with 10% methanol, 3% H2O2 in phosphate-buffered saline (PBS) for 10 min to block endogenous peroxidase activity.

After this, sections were treated for 1 h with 5% normal donkey serum (NDS) (Jackson ImmunoResearch Laboratories, West Grove, PA, USA) in PBS with 0.2% Triton-X100 (Sigma-Aldrich, St Louis, MO, USA) and were incubated overnight at room temperature in polyclonal rabbit anti-p75NTR antibody (1:1000, Alomone Labs). After washing, sections were incubated for 2 h with donkey anti-rabbit IgG biotinylated antibody (1:250; Jackson ImmunoResearch Laboratories, West Grove, PA, USA), followed by avidin–biotin–peroxidase complex (ABC; Vector Laboratories, Peterborough, UK) diluted in PBS, for 30 min. Colour development was achieved by incubating with 0.05% 3,3-diaminobenzidine tetrahydrochloride (Sigma-Aldrich) and 0.033% hydrogen peroxide in PB for 4 min. Finally, sections were mounted on slides, dried for one day at room temperature, dehydrated with ascending alcohols and rinsed in xylene. After this, sections were coverslipped using Eukitt mounting medium (PANREAC). All studied sections passed through all procedures simultaneously in order to minimize any difference from the immunohistochemical staining itself. To avoid any bias in the analysis, all slides were coded prior to analysis and remained so until the experiment was completed.

The antibodies which had been previously tested in their laboratory of origin, showed a regional and cellular immunolabelling similar to previous descriptions for these antigens. In order to exclude that some of the immunostaining was produced by the secondary antibodies or by the immunohistochemical protocol, primary antibodies were omitted or substituted by normal donkey serum. These controls resulted in a complete absence of immunostaining in every case.

## p75NTR and vAchT Positive Cells Quantification

The number of cells expressing the proteins p75NTR or vAchT (vesicular achetilcoline transporter) in the medial septum were quantified in 1, 6 and 14-month-old control and Ts65Dn mice using a modified version of the fractionator method [36], as previously described [37]. In brief, cells were counted covering 100% of the sample area, that is, within each section, all labelled cells were counted. The fractionator-sampling scheme refers to the methodology of examining one out of every six brain sections. Thus, our modification of the optical dissector combined with a 1:6 fractionator sampling is truly a modification of the optical fractionator method. 1:6 systematic-random series of sections covering the entire rostral to caudal extension of this structure were observed in an Olympus C×41 microscope under bright-field illumination, at 400× magnification under homogeneous illumination and photographs were taken with a CCD camera. The volume of the septal region analysed was determined for each animal using the Cavalieri’s principle [38]. To avoid any bias in the analysis, all slides were coded prior to analysis and remained so until the experiment was completed. After quantification, data were analysed with GraphPad Prism 9. In order to determine differences between groups, multiple unpaired *t*-test (parametric data, p75NTR) or Mann–Whitney *U* test (non parametric, vAchT) were used to assess the statistical significance (criterion at p < 0.05).

## Size of Cholinergic Neurons in the Medial Septum

We performed the size quantification of cholinergic neurons as described in previous studies [39]. In brief, the sections stained for p75NTR were used and the area of the cell bodies of at least 100 cells per animal (8 animals per group) were quantified using ImageJ software. The mean value for each group was compared using GraphPad Prism 9 software. In order to determine differences between groups (phenotype or ages), Multiple unpaired *t*-test were used to assess the statistical significance (criterion at p < 0.05).

## Single and Double Immunofluorescence

We performed single immunofluorescence against muscarinic receptor 1 for acetylcholine (mAchR1) to evaluate its hippocampal expression and double labelling (vAchT/c-fos) and (vAchT/FOXO1p) to evaluate respectively transcriptional activity and retention of the transcription factor FOXO1 in cholinergic neurons in the medial septum. Briefly, the general procedure for immunohistochemistry was followed but the peroxidase blocking step omitted. After washing, sections were incubated with citrate buffer (0.01 M, pH 6.0, 100 °C, 1 min) to recover antigenicity. Then, sections were treated for 1 h with 5% normal donkey serum (NDS) (Jackson ImmunoResearch Laboratories, West Grove, PA, USA) in PBS with 0.2% Triton-X100 (Sigma-Aldrich) and were incubated overnight at room temperature with polyclonal rabbit anti- mAchR1 (1:1000, Alomone, AMR-01) or with polyclonal goat anti-vAchT (1:5000, Millipore) with polyclonal rabbit anti c-Fos (1:1000, Santa Cruz biotechnology) or polyclonal rabbit anti FOXO1p (1:500, Cell Signaling). After washing, sections were incubated for 2 h with donkey anti-goat IgG conjugated with Alexa 488 (1:100, Invitrogen) and donkey anti-rabbit IgG conjugated with Alexa 555 (1:100, Invitrogen). After washing sections were mounted on microscope slides using a medium for fluorescent sections (Dako Citomation) and quantified using a confocal microscope Leica DM 2500.

## c-fos Expression in the Medial Septum Cholinergic Neurons

All the vAchT positive neurons in the medial septum of each animal were checked for c-fos expression. Sections were examined with a Leica DM25000 confocal microscope, homogeneously lighted and digitalized. Photographs were taken at 400× magnification and parallel subseries of Nissl stained sections were used as guidance to help to locate the region of interest. Confocal z-stacks covering the whole depth of the sections were taken with 1 μm step size and only subsets of confocal planes with the optimal penetration level for vAchT and c-fos antibodies were selected. Images were analysed using ImageJ software (NIH). A 5% intensity threshold (marked cells express c-fos at 95–100% relative intensity) was assessed and positive cells quantified. Collected data were analysed using GraphPad Prism 9 software. In order to evaluate differences between groups (phenotype or ages), Mann–Whitney U test was used to determine the statistical significance (criterion at p < 0.05).

## Senescence of Cholinergic Neurons

Double labelling of b-galactosidase activity in cholinergic neurons was performed. For senescence staining supplier specifications were followed. Briefly, sections were incubated at 37 °C with a solution containing (X-Gal stock solution 25 µL, 0,4 M potassium ferrycianide 12.5 µL, 0,4 M potassium ferrocianide 12.5 µL per ml of ultrapure water) during 4 h followed by PBS washes. Then samples were incubated with 10% NDS in PBS-Triton X-100 0.2% for 1 h and finally overnight with a solution containing polyclonal goat anti-vAchT (1:5000, Millipore). After washing, sections were incubated for 2 h with donkey anti-goat IgG conjugated with Alexa 555 (1:100, Invitrogen), washed again and mounted on microscope slides using a medium for fluorescent sections (Dako Citomation). Sections were examined for immunofluorescence for vAchT using a high sensitive Olympus DP71 camera attached to a fluorescence microscope Olympus BX41. Collected data was analysed using GraphPad Prism 9 software. In order to evaluate differences between groups (phenotype or ages), Mann–Whitney *U* test was used to determine the statistical significance (criterion at p < 0.05).

## Densitometrical Analysis of vAchT, mAchR1 and FOXO1p

For mAchR1 expression, a previously described methodology was used [40, 41]. Sections of hippocampus were examined with a Leica DM25000 confocal microscope, homogeneously lighted and digitalized. Photographs were taken at 400× magnification and parallel subseries of Nissl stained sections were used as guidance to help to locate the regions of interest. Confocal z-stacks covering the whole depth of the sections were taken with 1 μm step size and only subsets of confocal planes with the optimal penetration level for each antibody were selected. In order to avoid blood vessels and somata, small regions of the neuropil (505 μm^2^) were manually selected for analysis in these planes. Images were processed using ImageJ software as follows: the background was subtracted with a rolling value of 50, converted to 8-bit deep images and binarized using a determined threshold value. Then, the images were processed with a blur filter to reduce noise.

For the cellular expression of vAchT and FOXO1p in cholinergic neurons and vAchT expression in the neuropil of the hippocampus both control and trisomic mice of different ages were studied. For analysis of the intensity of the expression a previously described methodology was used [42]. Sections were examined with a Leica DM25000 confocal microscope under homogeneous illumination and digitalized. Photographs were taken at 400× magnification and images converted to grey level images that were measured using Image J software (NIH). For cellular quantification, fifty cells from each sample were analysed. Means were determined for each experimental group (Ts65Dn vs. euploid littermates and age (young, adult and old). Data has been analysed using GraphPad Prism 9 software. In order to evaluate differences between groups (phenotype or ages), Mann–Whitney *U* test was used to detrimne the statistical significance (criterion at p < 0.05) (for non parametric data) or Multiple unpaired *t*-test (criterion at p < 0.05) (for parametric data).

## Results

**Fig. 1:**
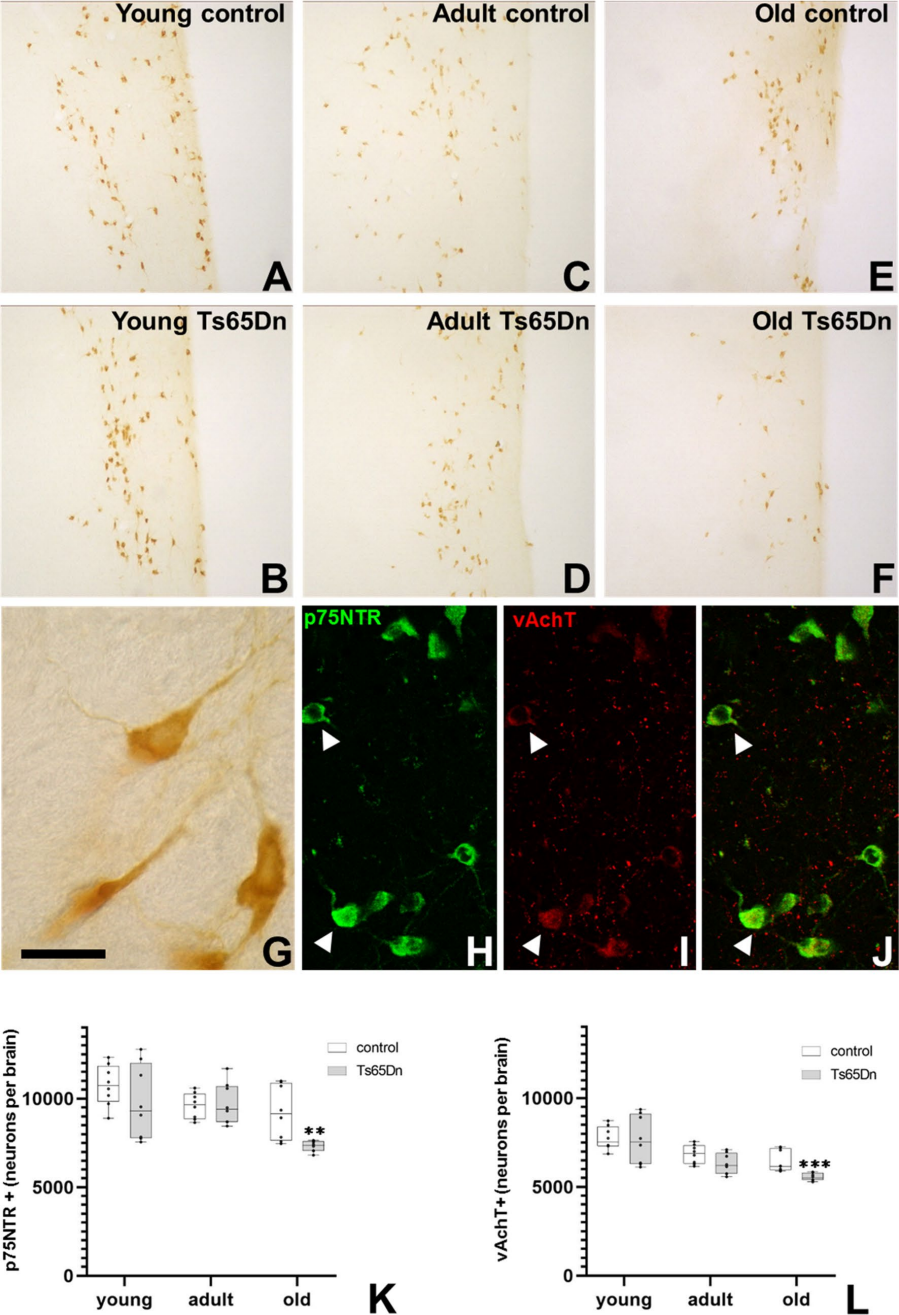
Loss of cholinergic neurons in the medial septum of old Ts65Dn mice. A-F. Representative images of the expression of p75NTR in the medial septum of control (**A**, **C**, **E**) and Ts65Dn mice (**B**, **D**, **F**) of different ages (young, **A**, **B**; adult **C**, **D**; old **E**, **F**). **G** Detail of p75NTR neurons in the medial septum; note the staining covering the entire cell. **H**–**J** Confocal images of double immunohistochemistry for p75NTR (green) and vAchT (red) in the medial septum of a control mouse (arrowheads indicate double labelled neurons). **K** Graph representing the mean value of total cells expressing p75NTR in the medial septum at different ages in control (white box) and Ts65Dn (grey box) mice. **L** Graph representing the mean value of total cells expressing vAchT in the medial septum at different ages in control and Ts65Dn mice. (**p < 0.01; ***p < 0.001). Scale bar represents 200 µm **A**–**F**; 20 µm G; 100 µm, **H**–**J**

In order to define the temporal profile of loss of cholinergic neurons in the medial septum, we examined the number of cholinergic neurons in control and trisomic mice of different ages using the receptor p75NTR and vAchT as markers (Fig. 1). As previously reported [43], and shown in Fig. 1A–G, p75NTR marker defines perfectly the morphology of cholinergic neurons. Analysis of the expression of p75NTR showed a statistically significant reduction in the number of cholinergic neurons between young-adult trisomic mice and old (14-month-old) trisomic mice (9222 ± 537 vs 7909 ± 100 cholinergic neurons, p = 0.0051). Control animals did not present this reduction between adult and old mice (Fig. 1K). Moreover, analysing every group, there was no difference between phenotypes except in old mice where fewer cholinergic neurons were found in trisomic mice as compared to their euploid littermates. Cholinergic neurons express the vAchT protein as well as p75NTR. We observed that some cholinergic neurons presented a low expression of vAchT compared to p75NTR, thus resulting in a lower number of vAchT positive cells (Fig. 1H–J). The analysis of the number of cells expressing vAchT neurons in the medial septum resulted in a similar pattern of expression with only a statistically significant difference between old control and trisomic mice (6462 ± 214 vs. 5549 ± 73 p = 0.00016).

**Fig. 2:**
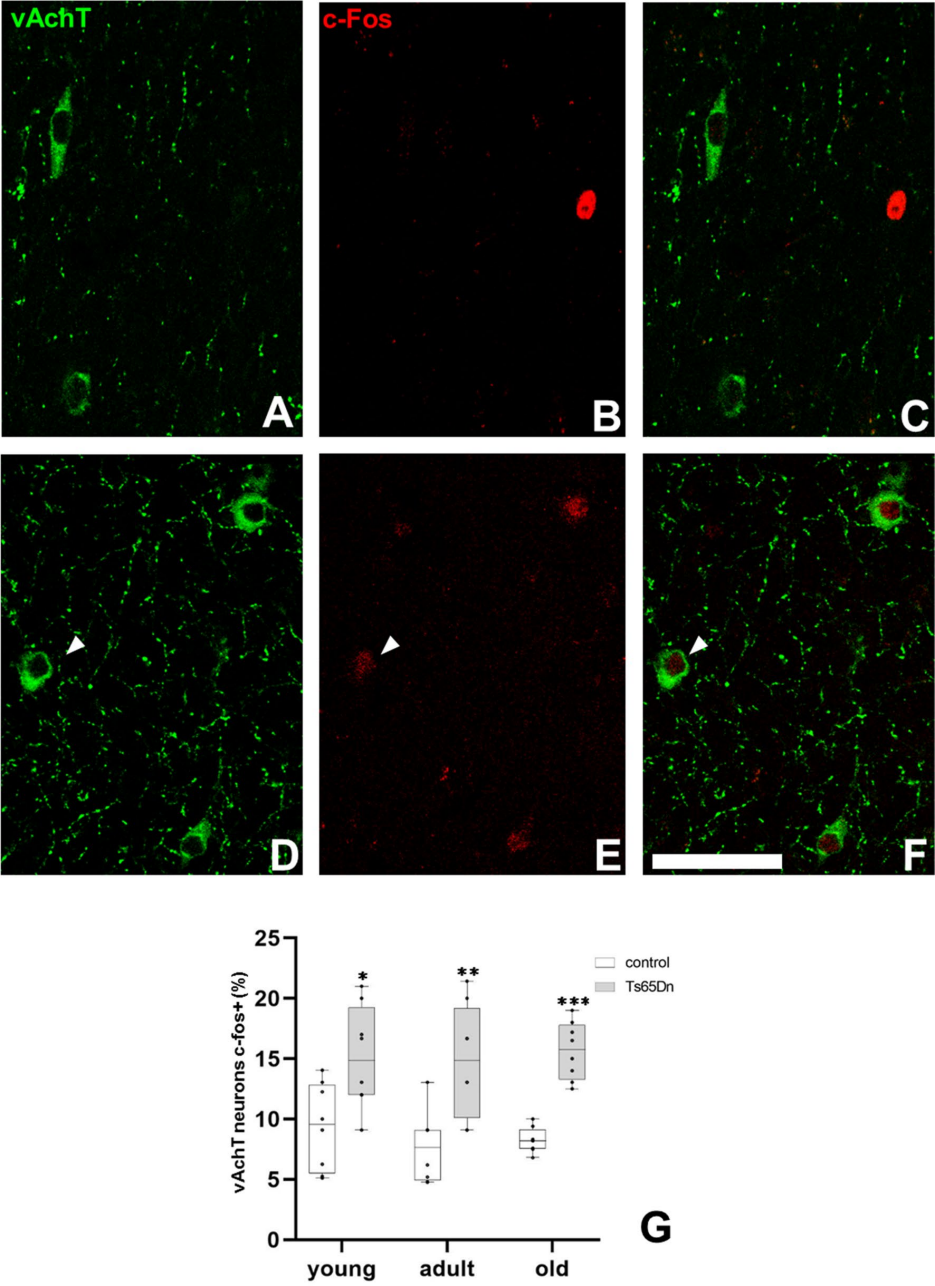
Cellular activation of cholinergic neurons in the Ts65Dn mouse model. **A**–**F** Confocal images showing the expression of vAchT (green), c-fos (red) and co-localization of both markers (arrowhead indicates a double labelled neuron). **G** Graph representing the mean value of the percentage of cholinergic neurons positive for c-fos at different ages in control (white box) and Ts65Dn (grey box) mice. (*p < 0.05; **p < 0.01; ***p < 0.001). Scale bar represents 100 µm

In the following step, the activity status of those cells was analysed by performing double labelling for the vesicular transporter for acetylcholine, vAchT (expressed by cholinergic neurons) and the early expression gene c-fos (Fig. 2). We quantified the percentage of cholinergic neurons expressing c-fos (over a set threshold). Expression of the vAchT delimitated clearly the cholinergic neurons (Fig. 2A–F), similar to what we had observed previously with p75NTR. Quantification of cholinergic neurons positive for c-fos at different ages in control and trisomic mice revealed that the percentage of active cells remained stable in each phenotype (Fig. 2D). However, the percentage of active cholinergic neurons was higher in trisomic mice as compared to control littermates in every age analysed (around 10 vs. 15%) (Fig. 2G).

**Fig. 3:**
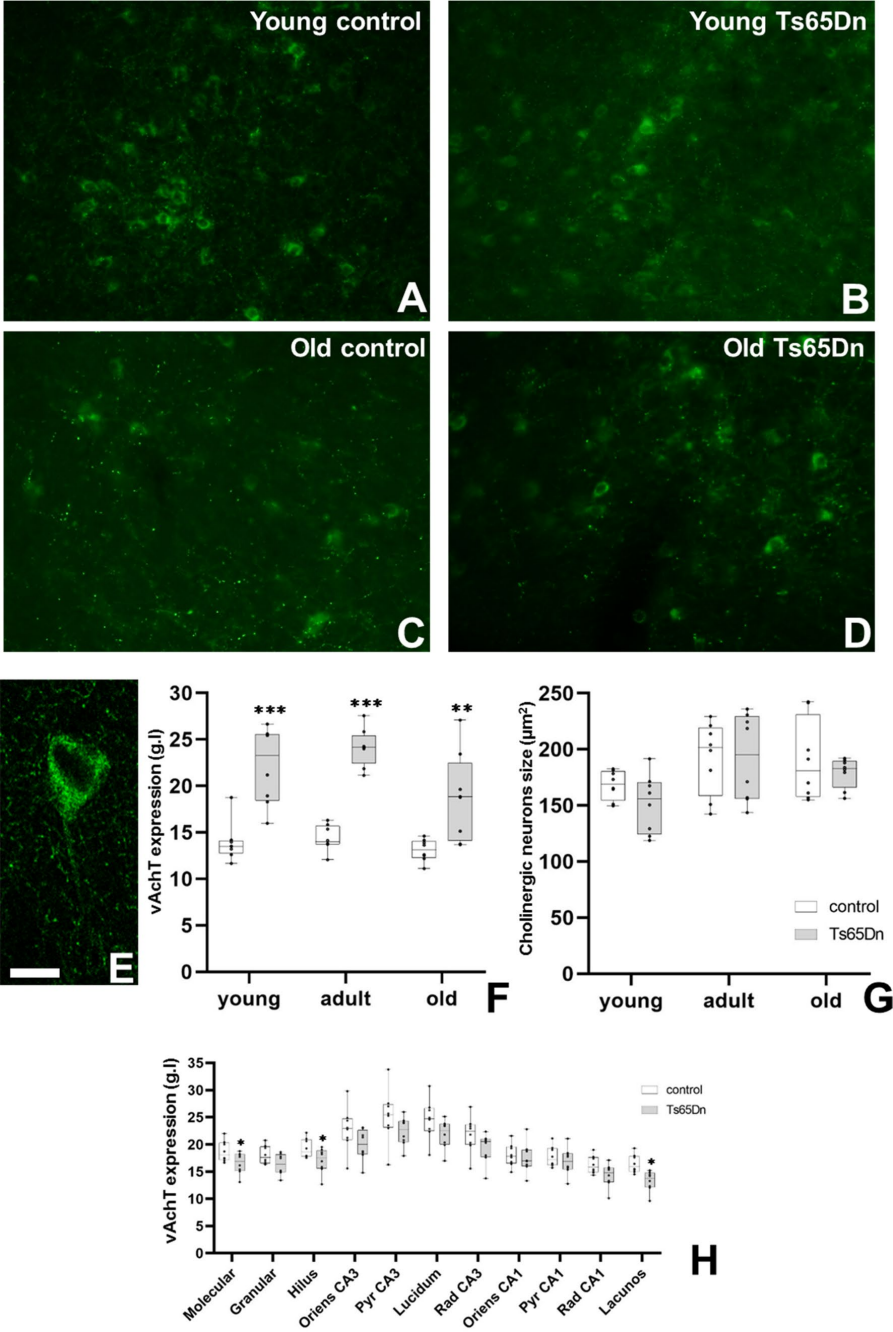
Expression of vAchT in cholinergic neurons in the Ts65Dn mouse model. **A**–**D** Representative images of vAchT expression in cholinergic neurons in young **A**–**B** and old **C**–**D** mice. **E** Detail of the expression of vAchT in the cytoplasm of a cholinergic neuron. **F** Graph representing the mean value of the intensity of expression of vAchT (grey levels) in cholinergic neurons of different ages in control (white box) and Ts65Dn (grey box) mice. **G** Graph representing the mean value of the size of cholinergic neurons (in µm^2^) in cholinergic neurons of different ages in control (white box) and Ts65Dn (grey box) mice. **H** Graph representing the mean value of the intensity of expression of vAchT (grey levels) in the different subregions of the hippocampus of old control (white box) and Ts65Dn (grey box) mice. (*p < 0.05; **p < 0.01; ***p < 0.001). Scale bar represents 100 µm (**A**–**D**) and 20 µm in (**E**)

**Fig. 4:**
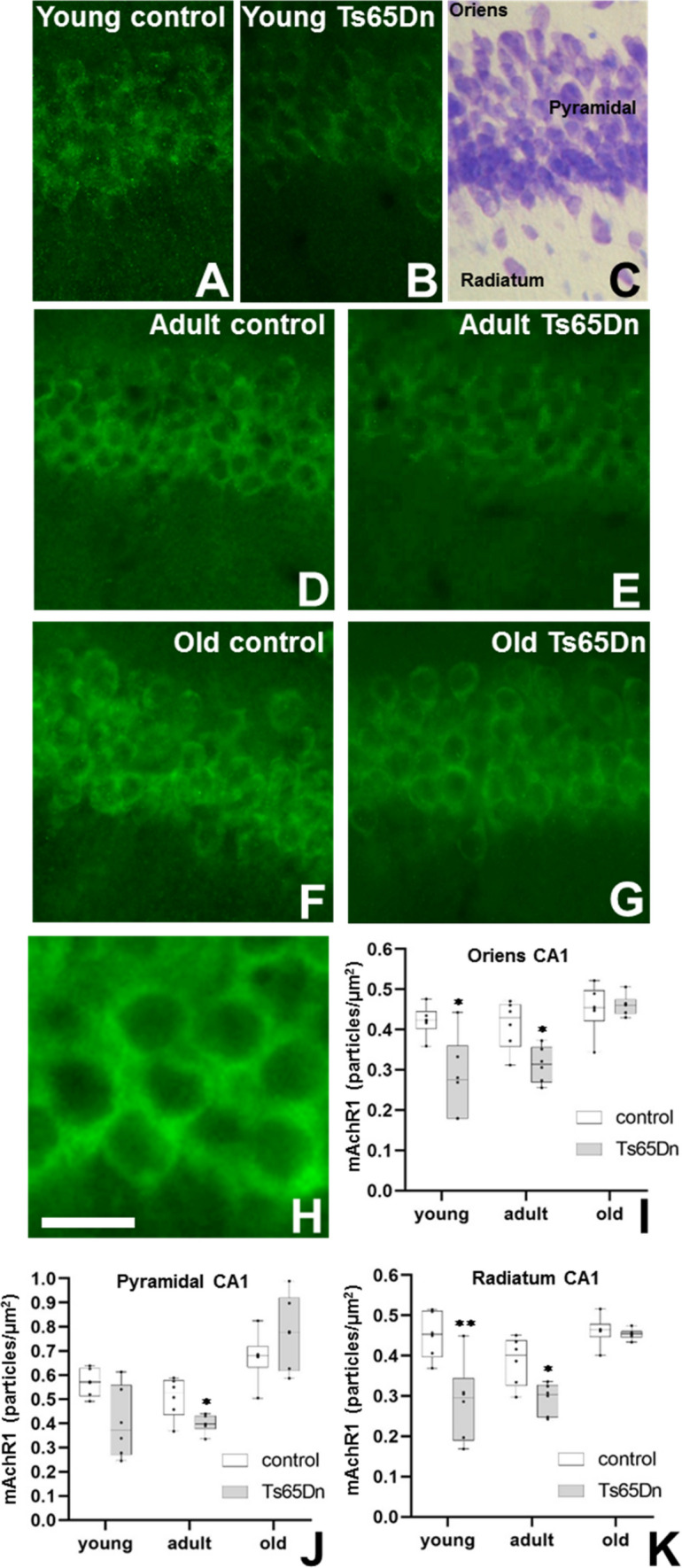
Expression of muscarinic receptor 1 (mAchR1) in the CA1 region of the hippocampus of the Ts65Dn model. Representative images of the expression of mAchR1 in control (**A**, **D**, **F**) or trisomic mice (**B**, **E**, **G**) of different ages. **C** Nissl stained section adjacent to the region analysed. **H** Detail of the expression of this receptor in pyramidal CA1 neurons. Graphs showing the density of expression (particles/ µm^2^) of mAchR1 in Oriens (**I**), Pyramidal (**J**) and Radiatum (**K**) of CA1. (*p < 0.05; **p < 0.01) in control (white box) and Ts65Dn (grey box) mice. Scale bar represents 50 µm (**A**–**G**) and 20 µm in **G**

After observing the elevated activity of cholinergic neurons in trisomic mice, and in order to determine the acetylcholine content in these cells, we analysed the intensity of vAchT expression in single cholinergic neurons (Fig. 3). We detected an excess of vAchT expression in cholinergic neurons of trisomic mice as compared to controls (Fig. 3A–E). As a general feature, we found a slight reduction of expression of vAchT with age in both, control and trisomic mice. However, the expression of vAchT was higher in trisomic mice in all age groups analysed (Fig. 3F) without changes among ages. Changes in the expression of this marker could possibly be related to alterations in the size of cholinergic neurons. Analysing the size of cholinergic neurons in the medial septum of both phenotypes and different ages we observed almost no change in size between groups and ages (Fig. 3G); therefore changes in the expression must be related to changes in its metabolism rather than in its size. Next, we studied the main projection region of medial septum cholinergic neurons (the hippocampus) focussing on the expression of vAchT and mAchR1 (Fig. 4). For vAchT, we analysed the expression of this marker in the neuropil of adult control and trisomic mice. In general, we observed no changes in the expression of adult mice. Only strata moleculare, hilus and lacunosum displayed a reduction in the expression of this marker (Fig. 3H). As for m1AchR1, we observed a similar pattern of change in the entire hippocampus; here we show the results in the CA1 area as an example. Expression of mAchR1 was especially prominent in the cell bodies of principal neurons and in the principal dendrites (Fig. 4A–H). Comparison of the expression intensity of this receptor between phenotypes and ages showed that control animals maintained the same expression level over age in all the regions analysed. However, young and adult trisomic mice (where cholinergic neurons were present and over-activated) displayed less mAchR1 expression as compared to old trisomic animals (where cholinergic neuron number diminished). Surprisingly, we observed a similar level of mAchR1 expression in old control and old trisomic mice (Fig. 4I–K).

**Fig. 5:**
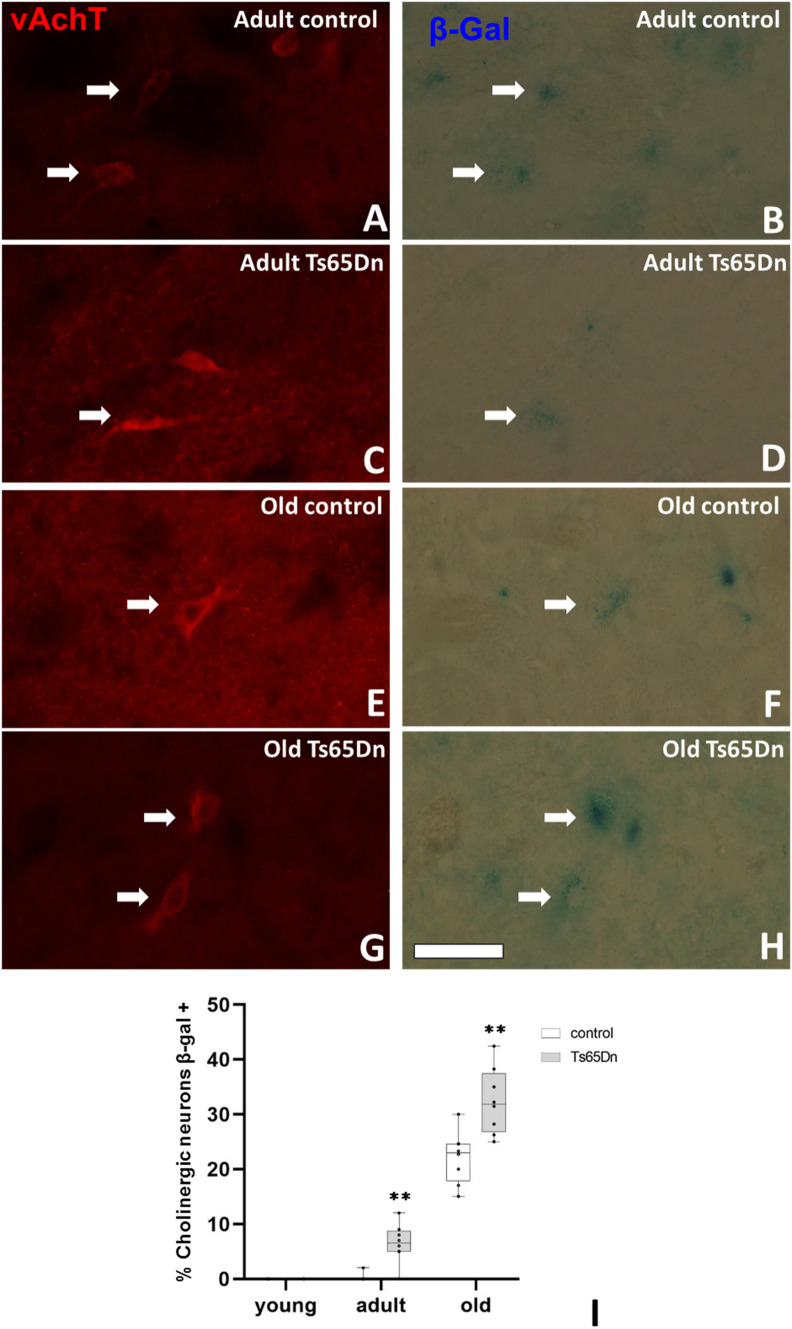
Senescence of cholinergic neurons in the Ts65Dn mouse model. Representative images of the presence of the senescent marker β-gal (blue) in cholinergic neurons, vAchT (red) in adult control (**A**–**B**), adult Ts65Dn (**C**–**D**), old control (**E**–**F**) and old Ts65Dn (**G**–**H**) mice (arrows indicated double labelled neurons). **I** Graph representing the mean value of the percentage of the presence of β-gal in cholinergic neurons in control (white box) and Ts65Dn (grey bar) mice. (**p < 0.01). Scale bar represents 50 µm

Since we observed a progressive loss of cholinergic neurons but an excess of cellular activity (observed by c-fos expression and vAchT expression) coupled to parallel changes in the expression of mAchR1 in the hippocampus, we decided to study the possible mechanism underlying cholinergic neuron degeneration in trisomic mice. Since we failed to find apoptotic cholinergic neurons (data not shown) and previous studies pointed to the possibility of an increase in senescent cells in trisomic mice [44], we decided to investigate the senescent state of these neurons. Samples from control and trisomic mice of different ages were double-labelled with β-galactosidase and vAchT. In adult and old mice β-galactosidase staining was present in cholinergic neurons, whereas none was detected in young mice (Fig. 5A–H). Statistical analysis of the percentage of cholinergic neurons displaying β-galactosidase staining (senescent cholinergic neurons) (Fig. 5I) revealed a significant difference dependent of phenotype (p < 0.05) and age (p < 0.001). Separate analysis by groups demonstrated clearly the presence of more senescent cholinergic neurons of both, adult and old trisomic mice (in percentage adult: 0.25 ± 0.25 vs. 6.50 ± 1.24 p = 0.0014; old: 22.18 ± 1.67 vs. 32.37 ± 2.28 p = 0.0011).

**Fig. 6:**
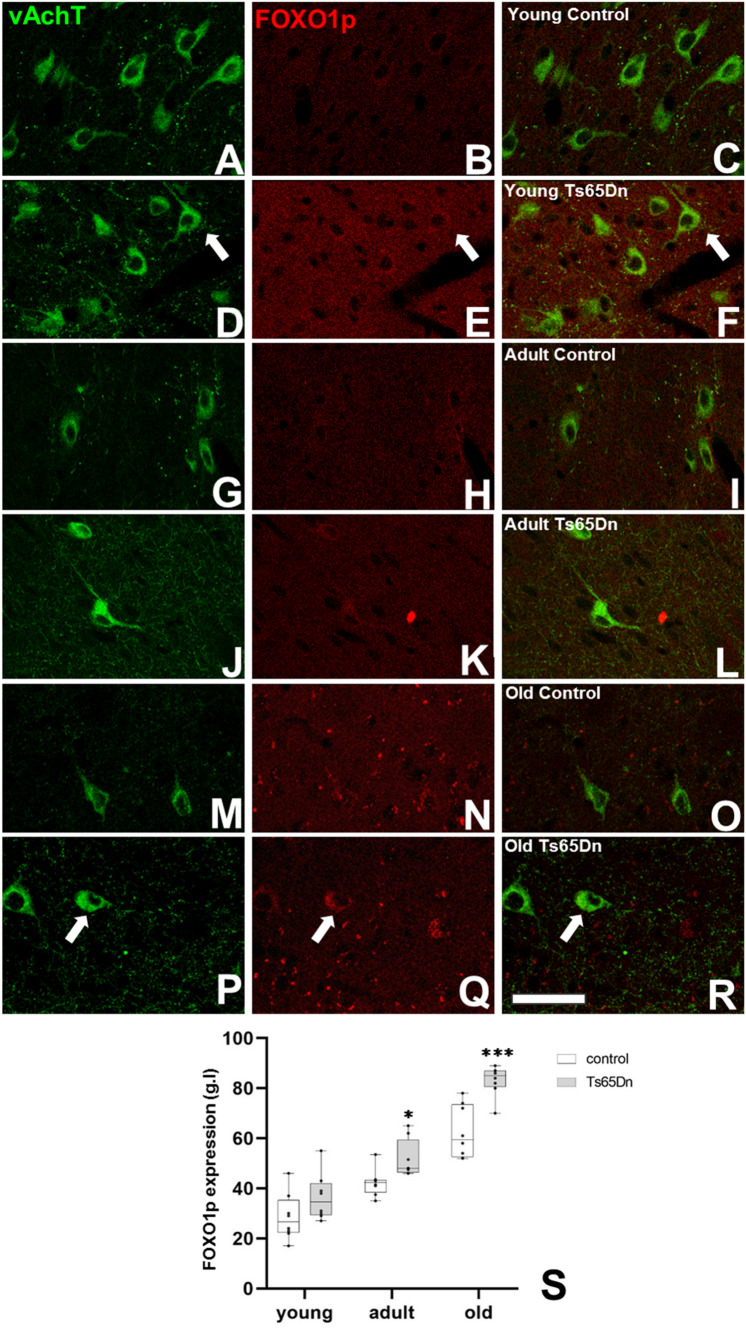
Alteration in the expression of the phosphorylated form of the FOX1 transcription factor in cholinergic neurons in the Ts65Dn mouse model. Representative images of the presence of pFOXO1 (in red) in cholinergic neurons, vAchT (in green) in young control (**A**–**C**), young Ts65Dn (**D**–**F**), adult control (**G**–**I**), adult Ts65Dn (**J**–**L**), old control (**M**–**O**) and old Ts65Dn (**P**–**R**) mice (arrows indicated double labelled neurons). **S** Graph representing the intensity of expression of pFOXO1 (grey levels) in the cytoplasm of cholinergic neurons in control (white box) and Ts65Dn (grey box) mice. (*p < 0.05; ***p < 0.001). Scale bar represents 75 µm

Previous studies have shown over-activation of the Akt pathway in trisomic mice [45]. Since the phosphorylated form of the FOXO1 transcription factor forms part of this pathway and has been related to senescence induction (or apoptosis blocking), we examined the expression of FOXO1p retained in the cytoplasm of cholinergic neurons (Fig. 6A–R). In general, a progressive increase of cytoplasmic FOXO1p in cholinergic neurons with age was observed, whereas no difference between young control and Ts65Dn mice could be found. However, adult and old trisomic mice displayed a higher expression of FOXO1p in the cytoplasm of cholinergic neurons as compared to their euploid littermates (in grey levels adult: 42.3 ± 1.9 vs. 51.8 ± 2.6 p = 0.0034; old: 62.6 ± 3.7 vs. 83.1 ± 2.1 p = 0.0011) (Fig. 6S).

## Discussion

In this study, we have quantified the number of cholinergic neurons in the medial septum of control and Ts65Dn mice of different ages (1, 6 and 14-months-old) using two different markers (p75NTR and vAchT) and obtaining similar results. We observe a general reduction in the number of medial septum cholinergic neurons with age but in particular, a greater loss in old Ts65Dn mice. Using the early expressing gene c-fos as an indicator for functional cholinergic neurons, we find that Ts65Dn cholinergic neurons display an excess of activation and an overexpression of vAchT without changes in size. The main target of the cholinergic neurons in the medial septum is the hippocampus. We observe a slight reduction of vAchT expression in some regions of the hippocampus of old trisomic mice. Analysis of mAchR1 expression in the hippocampus shows that the expression of the receptor does not change with age in control mice, however in old trisomic mice we observe an increase of its expression accompanied by an increase in the degenerative process. Due to the lack of cholinergic neurons displaying apoptotic markers, we have analysed the senescent state by using β-galactosidase expression, a reliable marker of senescence. We observe a clear increase of senescent cholinergic neurons in the medial septum of trisomic mice. A possible mechanism for senescence induction and apoptosis inhibition could be the phosphorylation of the transcription factor FOXO1, a member of the signalling Akt signalling cascade. In fact, we observe an excess of FOXO1p in the cytoplasm of cholinergic neurons of old trisomic mice. Most likely, the functional excess found in cholinergic neurons generates a process of early senescence that culminates in the loss of these neurons.

The use of p75NTR as a marker of cholinergic neurons is widely accepted [46]. Previous studies have analysed the cholinergic profile in human samples from DS patients as well as in the Ts65Dn murine model for DS. As a general observation, these studies have shown a normal expression pattern of the p75NTR in samples from young humans or mice and a reduction in samples from old individuals [13, 18, 47, 48]. Here we find less p75NTR expressing neurons in old mice as compared to young and adult ones, thus suggesting a neurodegenerative process especially in 14-months-old trisomic mice. Our data correlate with previous studies that have reported a reduction of cholinergic neurons between 6 and 10 months [13, 47]. Nevertheless, other studies show that the reduction is only significant at 20 months [12], at 12 months [18] or at 18–19 months of age [43]. In order to ensure that the reduction in the number of medial septum cholinergic neuron is not due to a reduction of the receptor for neurotrophin, we have studied the number of cholinergic neurons using vAchT as an alternative marker, and found similar results.

Colocalization studies between vAchT and c-fos allowed us to determine the activation state of cholinergic neurons in the medial septum in control and Ts65Dn mice, showing statistically significant differences for genotypes of all ages. Previous studies that reported an over-activation of neurons in individuals with DS [49] as well as in Ts65Dn mice [50] support these results. Moreover, studies of our group (Carbonell et al., in prep.) show an over-expression of c-fos by neurons located in the piriform cortex. Interestingly, this over-activation does not take place in all cells in individuals with DS; in fact, studies in DS lymphocytes have shown a reduction of c-fos [51]. One possible explanation could be that the over-function of cholinergic neurons in old trisomic mice is a consequence of a decrease in their number. However, this phenomenon starts before the onset of neurodegeneration, giving way to an alternative explanation, in which the over-activation could be responsible for the premature neuronal death observed in trisomic mice.

In order to analyse whether the over-activation correlates with the normal functioning of cholinergic neurons we have quantified the expression of vAchT in control and trisomic mice of different ages. We find a progressive decrease in the expression of vAchT with age in both groups. Interestingly, in trisomic mice we observe an over-expression of vAchT in all ages. Overexpression of vAchT has been correlated to increase acetylcholine concentration and related to accelerated aging processes [52]. This alteration supports previous studies that have shown an increase in choline acetyl transferase (ChAT) activity in 6-months-old [18]; 10-months-old [43] and 12-months-old [53] Ts65Dn mice. Moreover, patients in early stage of AD development display an increase in cholinergic activity, suggesting a compensatory response [54]. All these data suggest that cholinergic neurons are over-activated and over-functioning beginning early in life. Moreover, this over-activation could be the basis of the posterior neurodegeneration. Changes in the size of cholinergic neurons could be responsible, at least in part of the different somatic expression of vAchT. For this reason, we have analysed the size of medial septum cholinergic neurons and we did not observe changes with age or phenotype. This results support the idea of an alteration in the expression of the transporter rather than structural changes.

These results seem to contradict the classic cholinergic hypothesis [20] that relates the hypofunction of the cholinergic system to the impairment in the cognitive profile. In our Ts65Dn mouse model learning and memory impairment starts at 6-months of age [13, 47], whereas in other models the cholinergic degeneration starts later, at around 14 months or 20 months [24].

Recently, Dasari and Gulledge (2011) [55] showed that mAchR1 together with receptor m4 are responsible for the cholinergic modulation of excitatory circuits in the hippocampus, the main target of medial septum cholinergic neurons. More concretely, mAchR1 is involved in memory consolidation [56]. Analysing the expression of mAchR1 in the hippocampus we observe a reduction in the expression of this receptor in the young and adult trisomic hippocampal region CA1 and surprisingly an increase in its expression with age, noticeably between adult and old animals. Previous studies in individuals with DS have shown similar results [57] where the authors observed a progressive increase in the expression of the receptor with age in human trisomic samples. A possible explanation for this finding could be that the brain counteracts the decrease in acetylcholine by increasing the expression of the receptor and thus becoming more sensitive to the neurotransmitter. However, the early deficit we observe in the expression of the mAchR1 in young and adult trisomic mice must have a genetic origin or, perhaps, could be related to the hyperfunction of cholinergic neurons seen at those ages (Figs. 2, 3) and consequently to the possible excess of neurotransmitter in the media. Following this idea we have analysed the expression of vAchT in the different subregions of old trisomic mice (Fig. 3H). The CA1 region displays similar expression of vAchT between control and Ts65Dn mice correlating with similar expression of mAchR1 observed in these regions in old mice (Fig. 4I–K).

The degeneration of cholinergic neurons is a possible consequence of the reduced availability of NGF necessary for their survival [27]. Studies in human samples and Ts65Dn mice have revealed alteration in the processing of NGF giving rise to a low concentration of this factor [25]. Low concentration of NGF affects cholinergic neuron survival by inducing cellular apoptosis [58] mediated by cleaved caspase 3 [59]. Therefore, we set out to quantify the number of cholinergic neurons expressing the apoptotic marker caspase 3. We failed to observe cholinergic neurons undergoing apoptosis in any age group or phenotype. Since apoptosis is a short process, it is possible that we missed it in our samples. However, we neither observed apoptotic nor residual bodies in the region. The apoptotic extension in the brain of individuals with DS and murine models for this alteration is controversial. Some studies demonstrated an excess of apoptotic neurons in the brain of DS individuals as well as in some murine models [60]. However, other groups, among them ours, demonstrated no alteration or even reduction in the amount of apoptotic neurons in the brain of individuals with DS and the murine model Ts65Dn [61–63]. An interesting alternative could be that neurons instead enter a senescent state by blocking the apoptotic process [64] and consequently die through a non-apoptotic pathway.

Cellular senescence has emerged as a hallmark of AD (for a review see [65]). Recent studies have reconsidered its role in neurodegenerative diseases assuming a potential primary role in these pathologies where cellular senescence has been related to pro-inflammatory molecules [66]. In normal conditions, these senescent cells release pro-inflammatory molecules, as well as other signalling molecules thus stimulating the immune system to clear these senescent cells [67, 68]. However, when the immune system is not able to eliminate them [69], a large accumulation of senescent cells will trigger a chronic inflammatory state that gives rise to cellular damage. The factors that can induce cellular senescence are numerous such as DNA damage, reactive oxygen species, mitochondrial dysfunction, lysosomal alteration, amyloid deposition. Most of these factors are present in DS. Microglia and astrocytes are the effectors of the inflammatory response. Recent studies have shown alterations in microglia even in early stages [70] that could possibly induce an early senescence in neurons in DS and among them in cholinergic neurons. To test this hypothesis we have performed double labelling using vAchT antibodies and β-galactosidase staining (a reliable marker of senescence). We observe a normal increase in the cholinergic senescent cell number with age, but an acceleration of the senescence process in trisomic mice. This result is in accordance with previous studies in other brain regions of this model, such as the hippocampus [44] or the temporal cortex (Mateu et al., in prep). This senescence process could be mediated by the excess of ROS observed in individuals with DS and AD [71]. Oxidative stress is observed in individuals with DS starting early in development affecting neurogenesis, neuronal differentiation as well as survival of cholinergic neurons [44]. Mechanistically, previous studies have shown alterations in some signalling pathways that could explain our observations. Studies in the hippocampus of trisomic mice have demonstrated an increase in the amount of phosphorylated calcium calmodulin kinase II (pCAMKII) as well as the phosphorylated form of protein Kinase B also known as Akt (pAkt) [45]. In fact, it is known that Akt is recruited to the cell membrane where it is double phosphorylated and in turn phosphorylates the Forkhead family transcription factors (FOXO), among them FOXO1. Phosphorylation of FOXO1 inactivates its function as a pro-apoptotic transcription factor [72]. In our study, we observe an overexpression of the phosphorylated form of the FOXO1 that, together with the previously reported over-activation of Akt, could lead to an inhibition in FOXO1 function and subsequent inhibition of the apoptotic pathway. Our results are in accordance with this possibility as we observe a process of senescence rather than an apoptotic pathway in cholinergic neurons in the septum of trisomic mice.

Our results advocate a different picture of neurodegeneration of cholinergic neurons in the aged Ts65Dn mice. We suggest that an excess of cholinergic neuronal function induces a senescent pathway leading to a reduction in their number. Changes in the hippocampus, the main target of septal cholinergic neurons, accompany this process. The increased expression of specific receptors for acetylcholine, such is mAchR1; counteract the reduction of cholinergic innervation in the hippocampus. Even in aged mice, we fail to observe apoptotic cholinergic neurons. A possible explanation is that increased phosphorylation of Akt induces phosphorylation of the transcription factor FOXO1. We demonstrate that aged Ts65Dn mice have a greater number of cholinergic neurons with cytoplasmic-sequestered phosphorylated/inactivated FOXO1 transcription factor known to participate in its non-phosphorylated form in stress-related apoptosis. This observation could explain the lower apoptosis rate for cholinergic neurons, as well as other neurons in the brain of the Ts65Dn mice [61–63].

## Supplementary Information

Below is the link to the electronic supplementary material.

Supplementary file1 (DOCX 34 kb)
