# Supplementary material for: Cholinergic Senescence in the Ts65Dn Mouse Model for Down Syndrome
Source: Neurochem Res. 2022 Jun 29;47(10):3076–92. doi: 10.1007/s11064-022-03659-0 (PMC9470680; doi:10.1007/s11064-022-03659-0)
Supplement: Supplementary file 1 — Supplementary file1 (DOCX 34 kb) [file 11064_2022_3659_MOESM1_ESM.docx]

**STATISTICS**

**Number of p75 cells in the septum**

| **Normality** |  |  |  | **Homoscedasticity** |  |
| --- | --- | --- | --- | --- | --- |
| Shapiro-Wilk |  |  |  | Levene’s Test |  |
| W | gl | Sig. |  | F | Sig. |
| 0,957 | 48 | 0,079 |  | 2,692 | 0,108 |
|  |  | **>0.05** |  |  | **>0.05** |

We can use parametric tests

|  | Mean | SD | SE |
| --- | --- | --- | --- |
| young control | 10755 | 1157 | 409 |
| young Ts65Dn | 9762 | 2098 | 742 |
| adult control | 9603 | 733 | 259 |
| adult Ts65Dn | 9707 | 1175 | 416 |
| old control | 9222 | 1518 | 537 |
| old Ts65Dn | 7309 | 282 | 100 |

| **Multiple unpaired t-test** | | Discovery? | P value | Mean control | Mean Ts65Dn | Difference | SE of difference | t ratio | df | q value |
| --- | --- | --- | --- | --- | --- | --- | --- | --- | --- | --- |
|  | Young | No | 0,132861 | 10755 | 9762 | 992,8 | 647,7 | 1,533 | 42 | 0,201285 |
|  | Adult | No | 0,873814 | 9603 | 9707 | -103,5 | 647,7 | 0,1598 | 42 | 0,882553 |
|  | Old | **Yes** | **0,005119** | 9222 | 7309 | 1914 | 647,7 | 2,954 | 42 | **0,015512** |

**Number of vAchT positive cells in the septum**

| **Normality** |  |  |  | **Homoscedasticity** |  |
| --- | --- | --- | --- | --- | --- |
| Shapiro-Wilk |  |  |  | Levene’s Test |  |
| W | gl | Sig. |  | F | Sig. |
| 0,934 | 48 | 0,010 |  | 3,279 | 0,077 |
|  |  | **<0.05** |  |  | **>0.05** |

We can’t use parametric tests

|  | Mean | SD | SE |
| --- | --- | --- | --- |
| young control | 7736 | 647 | 228 |
| young Ts65Dn | 7664 | 1355 | 479 |
| adult control | 6844 | 522 | 184 |
| adult Ts65Dn | 6281 | 592 | 209 |
| old control | 6462 | 606 | 214 |
| old Ts65Dn | 5549 | 216 | 73 |

| **U Mann-Whitney** | Discovery? | P value | Mean rank of control | Mean rank of Ts65Dn | Mean rank diff. | Mann-Whitney U | q value |
| --- | --- | --- | --- | --- | --- | --- | --- |
| young | No | 0,720901 | 9,00 | 8,00 | 1,00 | 28 | 0,485407 |
| adult | No | 0,049883 | 10,88 | 6,125 | 4,75 | 13 | 0,050382 |
| old | **Yes** | **0,000155** | 12,5 | 4,5 | 8 | 0 | **0,000314** |

**Expression of c-fos in cholinergic neurons (%)**

| **Normality** |  |  |  | **Homoscedasticity** |  |
| --- | --- | --- | --- | --- | --- |
| Shapiro-Wilk |  |  |  | Levene’s Test |  |
| W | gl | Sig. |  | F | Sig. |
| 0,951 | 48 | 0,044 |  | 4,032 | 0,051 |
|  |  | **<0.05** |  |  | **>0.05** |

We can’t use parametric tests

|  | Mean | SD | SE |
| --- | --- | --- | --- |
| young control | 9,38 | 3,56 | 1,26 |
| young Ts65Dn | 15,1 | 4,22 | 1,49 |
| adult control | 7,66 | 2,92 | 1,04 |
| adult Ts65Dn | 14,88 | 4,61 | 1,63 |
| old control | 8,34 | 3,73 | 1,31 |
| old Ts65Dn | 15,66 | 2,38 | 0,84 |

| **U Mann-Whitney** | Discovery? | P value | Mean rank of control | Mean rank of Ts65Dn | Mean rank diff. | Mann-Whitney U | q value |
| --- | --- | --- | --- | --- | --- | --- | --- |
| **young** | **Yes** | **0,035742** | 6 | 11 | -5 | 12 | **0,037529** |
| **adult** | **Yes** | **0,00575** | 5,25 | 11,75 | -6,5 | 6 | **0,009056** |
| **old** | **Yes** | **0,000155** | 4,5 | 12,5 | -8 | 0 | **0,00049** |

**vAchT intensity in cholinergic neurons**

| Normality |  |  |  | Homoscedasticity |  |
| --- | --- | --- | --- | --- | --- |
| Shapiro-Wilk |  |  |  | Levene’s Test |  |
|  | gl | Sig. |  | F | Sig. |
| 0,861 | 48 | 0,000 |  | -5,986 | 0,000 |
|  |  | **<0.05** |  |  | **<0.05** |

We can’t use parametric tests

|  | Mean | SD | SE |
| --- | --- | --- | --- |
| young control | 13,93 | 2,1 | 0,74 |
| young Ts65Dn | 22,2 | 4,11 | 1,45 |
| adult control | 14,37 | 1,38 | 0,49 |
| adult Ts65Dn | 24,13 | 2,02 | 0,71 |
| old control | 13,09 | 1,17 | 0,41 |
| old Ts65Dn | 18,8 | 4,71 | 1,66 |

| **U Mann-Whitney** | Discovery? | P value | Mean rank of control | Mean rank of Ts65Dn | Mean rank diff. | Mann-Whitney U | q value |
| --- | --- | --- | --- | --- | --- | --- | --- |
| **young** | **Yes** | **0,000622** | 4,75 | 12,25 | -7,5 | 2 | **0,000979** |
| **adult** | **Yes** | **0,000155** | 4,5 | 12,5 | -8 | 0 | **0,00049** |
| **old** | **Yes** | **0,004662** | 5,25 | 11,75 | -6,5 | 6 | **0,004895** |

**Size of cholinergic neurons**

| Normality |  |  |  | Homoscedasticity |  |
| --- | --- | --- | --- | --- | --- |
| Shapiro-Wilk |  |  |  | Levene’s Test |  |
|  | gl | Sig. |  | F | Sig. |
| 0.967 | 48 | 0,194 |  | 0,023 | 0,879 |
|  |  | **>0.05** |  |  | **>0.05** |

We can use parametric tests

|  | Mean | SD | SE |
| --- | --- | --- | --- |
| young control | 168,3 | 12,8 | 4,5 |
| young Ts65Dn | 151,6 | 26 | 9,2 |
| adult control | 192,8 | 32 | 11,3 |
| adult Ts65Dn | 192,2 | 38,8 | 13,7 |
| old control | 189,7 | 35,9 | 12,7 |
| old Ts65Dn | 179,2 | 13,2 | 4,6 |

| **Multiple unpaired t-test** | Discovery? | P value | Mean control | Mean Ts65Dn | Difference | SE of difference | t ratio | df | q value |
| --- | --- | --- | --- | --- | --- | --- | --- | --- | --- |
| young | No | 0,127303 | 168,3 | 151,6 | 16,67 | 10,28 | 1,621 | 14 | 0,385728 |
| adult | No | 0,974281 | 192,8 | 192,2 | 0,5827 | 17,75 | 0,03282 | 14 | 0,984023 |
| old | No | 0,450357 | 189,7 | 179,2 | 10,52 | 13,55 | 0,7765 | 14 | 0,68229 |

**M1 expression in the hippocampal CA1 region**

|  | Normality |  |  | Homoscedasticity |  |
| --- | --- | --- | --- | --- | --- |
|  | Shapiro-Wilk |  |  | Levene’s Test |  |
|  |  | gl | Sig. | F | Sig. |
| orica1 | 0,919 | 36 | 0,012 | 9,831 | 0,004 |
| pyrca1 | 0,980 | 36 | 0,736 | 9,787 | 0,004 |
| radca1 | 0,914 | 36 | 0,008 | 7,502 | 0,010 |
|  |  |  | **<0.05** |  | **<0.05** |

We can’t use parametric tests

| **orica1** | Mean | SD | SE |  | **pyrca1** | Mean | SD | SE |  | **radca1** | Mean | SD | SE |
| --- | --- | --- | --- | --- | --- | --- | --- | --- | --- | --- | --- | --- | --- |
| young control | 0,422 | 0,038 | 0,015 |  |  | 0,57 | 0,057 | 0,023 |  |  | 0,451 | 0,057 | 0,023 |
| young Ts65Dn | 0,281 | 0,1 | 0,041 |  |  | 0,403 | 0,147 | 0,06 |  |  | 0,286 | 0,099 | 0,041 |
| adult control | 0,412 | 0,061 | 0,025 |  |  | 0,508 | 0,084 | 0,034 |  |  | 0,387 | 0,06 | 0,024 |
| adult Ts65Dn | 0,314 | 0,045 | 0,018 |  |  | 0,399 | 0,037 | 0,015 |  |  | 0,293 | 0,039 | 0,016 |
| old control | 0,452 | 0,06 | 0,025 |  |  | 0,676 | 0,102 | 0,042 |  |  | 0,462 | 0,037 | 0,015 |
| old Ts65Dn | 0,461 | 0,026 | 0,011 |  |  | 0,776 | 0,154 | 0,063 |  |  | 0,454 | 0,013 | 0,005 |

|  | **U Mann-Whitney** | |  |  |  |  |  |
| --- | --- | --- | --- | --- | --- | --- | --- |
| **orica1** | Discovery? | P value | Mean rank of control | Mean rank of Ts65Dn | Mean rank diff. | Mann-Whitney U | q value |
| **young** | **Yes** | **0,041126** | 8,667 | 4,333 | 4,333 | 5 | **0,062305** |
| **adult** | **Yes** | **0,025974** | 8,833 | 4,167 | 4,667 | 4 | **0,062305** |
| old | No | 0,937229 | 6,333 | 6,667 | -0,3333 | 17 | 0,946602 |
|  |  |  |  |  |  |  |  |
| **pyrca1** |  |  |  |  |  |  |  |
| young | No | 0,064935 | 8,5 | 4,5 | 4 | 6 | 0,098377 |
| **adult** | **Yes** | **0,041126** | 8,667 | 4,333 | 4,333 | 5 | **0,098377** |
| old | No | 0,393939 | 5,5 | 7,5 | -2 | 12 | 0,397879 |
|  |  |  |  |  |  |  |  |
| **radca1** |  |  |  |  |  |  |  |
| **young** | **Yes** | **0,008658** | 9,167 | 3,833 | 5,333 | 2 | **0,026234** |
| **adult** | **Yes** | **0,041126** | 8,667 | 4,333 | 4,333 | 5 | **0,062305** |
| old | No | 0,24026 | 7,833 | 5,167 | 2,667 | 10 | 0,242662 |

**βgal expression in cholinergic neurons**

| Normality |  |  |  | Homoscedasticity |  |
| --- | --- | --- | --- | --- | --- |
| Shapiro-Wilk |  |  |  | Levene's Test |  |
| w | gl | Sig. |  | F | Sig. |
| 0,772 | 48 | 0,000 |  | 3,727 | 0,060 |
|  |  | **<0.05** |  |  | **>0.05** |

We can’t use parametric tests

|  | Mean | SD | SE |
| --- | --- | --- | --- |
| young control | 0 | 0 | 0 |
| young Ts65Dn | 0 | 0 | 0 |
| adult control | 0,25 | 0,707 | 0,25 |
| adult Ts65Dn | 6,5 | 3,505 | 1,239 |
| old control | 22,18 | 4,723 | 1,67 |
| old Ts65Dn | 32,37 | 6,435 | 2,275 |

| U Mann-Whitney | Discovery? | P value | Mean rank of control | Mean rank of Ts65Dn | Mean rank diff. | Mann-Whitney U | q value |
| --- | --- | --- | --- | --- | --- | --- | --- |
| young | No | >0,999999 | 8,5 | 8,5 | 0 | 32 | 0,3367 |
| **adult** | **Yes** | **0,001399** | 5,063 | 11,94 | -6,875 | 4,5 | **0,0007** |
| **old** | **Yes** | **0,001088** | 4,875 | 12,13 | -7,25 | 3 | **0,0007** |

**Cytoplasmic expression of pFOXO1 in cholinergic neurons**

| Normality |  |  |  | Homoscedasticity |  |
| --- | --- | --- | --- | --- | --- |
| Shapiro-Wilk |  |  |  | Levene's Test |  |
| w | gl | Sig. |  | F | Sig. |
| 0,945 | 48 | 0,027 |  | 3,631 | 0,063 |
|  |  | **<0.05** |  |  | **>0.05** |

We can’t use parametric tests

|  | Mean | SD | SE |
| --- | --- | --- | --- |
| young control | 28,5 | 9,3 | 3,2 |
| young Ts65Dn | 36,5 | 9,3 | 3,3 |
| adult control | 42,3 | 5,4 | 1,9 |
| adult Ts65Dn | 51,8 | 7,4 | 2,6 |
| old control | 62,6 | 10,5 | 3,7 |
| old Ts65Dn | 83,1 | 6 | 2,1 |

| U Mann-Whitney | Discovery? | P value | Mean rank of control | Mean rank of Ts65Dn | Mean rank diff. | Mann-Whitney U | q value |
| --- | --- | --- | --- | --- | --- | --- | --- |
| young | Yes | 0,079254 | 6,375 | 10,63 | -4,25 | 15 | 0,0277 |
| **adult** | **Yes** | **0,003419** | 5,25 | 11,75 | -6,5 | 6 | **0,0018** |
| **old** | **Yes** | **0,001088** | 4,875 | 12,13 | -7,25 | 3 | **0,0011** |

**Hippocampal vAchT expression**

|  | Normality |  |  |  | Homoscedasticity |  |
| --- | --- | --- | --- | --- | --- | --- |
|  | Shapiro-Wilk |  |  |  | Levene's |  |
|  | W | gl | sig |  | F | Sig. |
| molec | 0,989 | 16 | 0,999 |  | 0,020 | 0,891 |
| gran | 0,989 | 16 | 0,999 |  | 0,816 | 0,382 |
| hilus | 0,968 | 16 | 0,800 |  | 0,360 | 0,558 |
| orica3 | 0,961 | 16 | 0,684 |  | 0,102 | 0,754 |
| pirca3 | 0,961 | 16 | 0,676 |  | 0,619 | 0,445 |
| lucidum | 0,979 | 16 | 0,956 |  | 0,255 | 0,622 |
| radca3 | 0,970 | 16 | 0,846 |  | 0,079 | 0,783 |
| orica1 | 0,977 | 16 | 0,936 |  | 0,305 | 0,589 |
| pirca1 | 0,958 | 16 | 0,626 |  | 0,433 | 0,521 |
| radca1 | 0,963 | 16 | 0,710 |  | 0,308 | 0,588 |
| lacunos | 0,973 | 16 | 0,890 |  | 0,021 | 0,887 |
|  |  |  | **>0.05** |  |  | **>0.05** |

We can use parametric tests

|  | Mean | SD | SE |
| --- | --- | --- | --- |
| Mol Control | 18,71 | 1,92 | 0,68 |
| Mol Ts65Dn | 16,56 | 1,97 | 0,69 |
| Gran Control | 18,08 | 1,68 | 0,59 |
| Gran Ts65Dn | 16,34 | 1,93 | 0,68 |
| Hilus Control | 19,27 | 1,78 | 0,63 |
| Hilus Ts65Dn | 17,03 | 2,29 | 0,81 |
| Orica3 control | 22,81 | 4,04 | 1,43 |
| Orica3 Ts65Dn | 19,9 | 2,87 | 1,01 |
| Pyrca3 control | 25,26 | 4,93 | 1,74 |
| Pyrca3 Ts65Dn | 22,36 | 2,66 | 0,94 |
| Lucidum control | 24,57 | 3,7 | 1,31 |
| Lucidum Ts65Dn | 21,86 | 2,66 | 0,94 |
| Radca3 control | 21,81 | 3,35 | 1,18 |
| Radca3 Ts65Dn | 19,34 | 2,77 | 0,98 |
| orica1 control | 18,04 | 2,1 | 0,74 |
| orica1 Ts65Dn | 17,51 | 2,78 | 0,98 |
| pyrca1 control | 17,75 | 1,91 | 0,67 |
| Pyrca1 Ts65Dn | 16,89 | 2,49 | 0,88 |
| radca1 control | 16,26 | 1,63 | 0,58 |
| radca1 Ts65Dn | 14,32 | 2,17 | 0,77 |
| lacunos control | 16,44 | 1,68 | 0,59 |
| lacunos Ts65Dn | 13,25 | 1,87 | 0,66 |

|  | Discovery? | P value | Mean of control | Mean of Ts65Dn | Difference | SE of difference | t ratio | df | q value |
| --- | --- | --- | --- | --- | --- | --- | --- | --- | --- |
| **molec** | **Yes** | **0,043813** | 18,71 | 16,56 | 2,153 | 0,9718 | 2,215 | 14 | **0,157** |
| gran | No | 0,074772 | 18,08 | 16,34 | 1,743 | 0,9052 | 1,925 | 14 | 0,157 |
| **hilus** | **Yes** | **0,046851** | 19,27 | 17,03 | 2,235 | 1,025 | 2,18 | 14 | **0,157** |
| orica3 | No | 0,119962 | 22,81 | 19,9 | 2,903 | 1,753 | 1,656 | 14 | 0,1724 |
| pirca3 | No | 0,164597 | 25,26 | 22,36 | 2,904 | 1,98 | 1,467 | 14 | 0,192 |
| lucidum | No | 0,114742 | 24,57 | 21,86 | 2,713 | 1,613 | 1,682 | 14 | 0,1724 |
| radca3 | No | 0,131351 | 21,81 | 19,34 | 2,463 | 1,537 | 1,603 | 14 | 0,1724 |
| orica1 | No | 0,671975 | 18,04 | 17,51 | 0,5323 | 1,231 | 0,4325 | 14 | 0,6414 |
| pirca1 | No | 0,451698 | 17,75 | 16,89 | 0,8602 | 1,111 | 0,7742 | 14 | 0,4743 |
| radca1 | No | 0,062491 | 16,26 | 14,32 | 1,945 | 0,9611 | 2,024 | 14 | 0,157 |
| **lacunos** | **Yes** | **0,002986** | 16,44 | 13,25 | 3,187 | 0,8889 | 3,585 | 14 | **0,0313** |
